# Clinical utility of maximum blink interval measured by smartphone application DryEyeRhythm to support dry eye disease diagnosis

**DOI:** 10.1038/s41598-023-40968-y

**Published:** 2023-08-21

**Authors:** Kenta Fujio, Ken Nagino, Tianxiang Huang, Jaemyoung Sung, Yasutsugu Akasaki, Yuichi Okumura, Akie Midorikawa-Inomata, Keiichi Fujimoto, Atsuko Eguchi, Maria Miura, Shokirova Hurramhon, Alan Yee, Kunihiko Hirosawa, Mizu Ohno, Yuki Morooka, Akira Murakami, Hiroyuki Kobayashi, Takenori Inomata

**Affiliations:** 1 Department of Ophthalmology, Juntendo University Graduate School of Medicine, 2-1-1 Hongo, Bunkyo-ku, Tokyo, 113-0033 Japan; 2 Department of Digital Medicine, Juntendo University Graduate School of Medicine, Tokyo, Japan; 3 Department of Hospital Administration, Juntendo University Graduate School of Medicine, Tokyo, Japan; 4 AI Incubation Farm, Juntendo University Graduate School of Medicine, Tokyo, Japan

**Keywords:** Eye diseases, Corneal diseases, Eye manifestations

## Abstract

The coronavirus disease (COVID-19) pandemic has emphasized the paucity of non-contact and non-invasive methods for the objective evaluation of dry eye disease (DED). However, robust evidence to support the implementation of mHealth- and app-based biometrics for clinical use is lacking. This study aimed to evaluate the reliability and validity of app-based maximum blink interval (MBI) measurements using DryEyeRhythm and equivalent traditional techniques in providing an accessible and convenient diagnosis. In this single-center, prospective, cross-sectional, observational study, 83 participants, including 57 with DED, had measurements recorded including slit-lamp-based, app-based, and visually confirmed MBI. Internal consistency and reliability were assessed using Cronbach’s alpha and intraclass correlation coefficients. Discriminant and concurrent validity were assessed by comparing the MBIs from the DED and non-DED groups and Pearson’s tests for each platform pair. Bland–Altman analysis was performed to assess the agreement between platforms. App-based MBI showed good Cronbach’s alpha coefficient, intraclass correlation coefficient, and Pearson correlation coefficient values, compared with visually confirmed MBI. The DED group had significantly shorter app-based MBIs, compared with the non-DED group. Bland–Altman analysis revealed minimal biases between the app-based and visually confirmed MBIs. Our findings indicate that DryEyeRhythm is a reliable and valid tool that can be used for non-invasive and non-contact collection of MBI measurements, which can assist in accessible DED detection and management.

## Introduction

Dry eye disease (DED) is the most commonly occurring ocular surface disease, affecting 5–50% of the population globally^1,2^. Its prevalence is expected to increase with the ongoing digitalization and aging of the society^2,3^. Patients with DED present with a wide range of symptoms, such as ocular pain, discomfort, and decreased visual acuity caused by decreased tear film breakup time (TFBUT) and kerato-conjunctival epithelial defects^4,5^. Hence, DED has a negative impact on productivity and quality of vision, thereby impacting quality of life and resulting in economic loss^6,7^. A significant proportion of patients with DED may be undiagnosed and do not seek treatment despite experiencing symptoms^8^, indicating the need for a novel approach that can expand the reach of DED screening, promote early diagnosis and intervention for the prompt management of symptoms, prevent decreased quality of life, and reduce the societal costs of DED management^5^.

The demand for non-invasive and non-contact examinations and the incorporation of telemedicine in routine practice have rapidly increased with the novel coronavirus disease (COVID-19) pandemic^9,10^. DED is diagnosed by evaluating subjective symptoms and objective findings on examinations, such as TFBUT and ocular surface staining^11,12^. Dry eye examinations require specialized equipment, such as slit-lamp microscopes and fluorescein dye; moreover, the invasive nature of the examination disrupts the true in vivo tear composition^12^. Therefore, performing a comprehensive DED evaluation in a telehealth setting is impractical, warranting the formal appraisal of various telehealth strategies to remotely diagnose DED and manage its symptoms^5,13,14^.

The maximum blink interval (MBI), which is defined as the duration that participants can keep their eyes open before blinking during each trial, correlates positively with TFBUT^15^. MBI can be measured non-invasively, without contact, under observation with a slit-lamp microscope. The combined use of a slit-lamp microscope and DED-specific symptom questionnaire has shown a sensitivity and specificity of 75.4% and 92.9%, respectively, for DED diagnosis^16^. By eliminating the requirement for slit-lamp-based MBI measurements, MBI could replace TFBUT in remote settings to enable non-invasive and non-contact DED diagnosis and monitoring.

In November 2016, we developed and released an in-house smartphone application (app), DryEyeRhythm, which is capable of measuring MBI and administering DED-specific symptom questionnaires^14,17,18^ with positive and negative predictive values, sensitivity, and specificity of 91.3%, 69.1%, 50.0%, and 95.0%, respectively^5^. DryEyeRhythm could measure MBI by biosensing blinking using smartphone-attached cameras. Additionally, recent data on various DED subtypes suggest that MBI monitoring is useful in determining the disease mechanism for stratified and personalized treatment approaches^18^. The administration of DED-specific questionnaires through DryEyeRhythm provides reliable patient-reported data^14,17^; however, MBI measurement through the app must be assessed for reliability and validity.

Therefore, in this study, MBI data that were collected through the DryEyeRhythm app (app-based MBI) were formally evaluated for their validity, reliability, and equivalence to visually confirmed MBI.

## Results

### Participants’ characteristics

**Figure 1:**
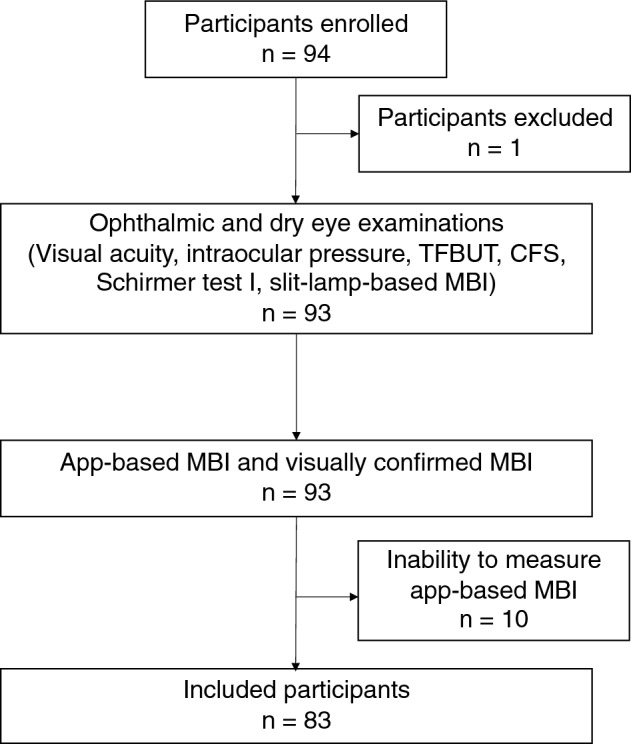
Flowchart of the study indicating the number of participants at each. CFS, corneal fluorescein staining; TFBUT, tear film breakup time; MBI, maximum blink interval.

Figure 1 shows the enrollment process. This study initially included 94 participants. One patient was excluded due to refusal to participate after the MBI measurement. Among the 93 remaining participants, 10 (10.8%) were excluded owing to their inability to obtain app-based MBI measurements. MBI was unobtainable in 10 participants—on both platforms in 4 participants, on the iPhone operating system (iOS) in 2 participants, and on Android in 4 participants. Table 1 presents the characteristics of the 83 included participants. Supplementary Table S1 shows a comparison between the included and excluded individuals.

**Table 1:** Participants’ characteristics.

Characteristics	Non-DED	DED	*P* value	Total
	n = 32	n = 51		n = 83
Age, year ± SD	61.4 ± 14.9	62.5 ± 12.0	0.855	62.0 ± 13.1
Sex, female (%)	31 (96.9)	47 (92.2)	0.379	78 (94.0)
J-OSDI, 0–100 ± SD	17.9 ± 21.4	36.0 ± 16.9	< 0.001	29.1 ± 20.6
TFBUT, s ± SD	4.5 ± 2.6	2.6 ± 1.0	< 0.001	3.4 ± 2.0
CFS, 0–9 ± SD	2.7 ± 2.5	3.1 ± 2.5	0.282	2.9 ± 2.5
Schirmer I, mm ± SD	8.6 ± 7.6	7.2 ± 7.9	0.235	7.7 ± 7.7
Slit-lamp-based MBI, s ± SD	12.4 ± 7.0	9.6 ± 6.6	0.035	10.7 ± 6.7

*P* values were determined using a two-tailed Student’s t test for continuous variables and χ2 test for categorical variables. DED, dry eye disease; SD, standard deviation; J-OSDI, Japanese version of the Ocular Surface Disease Index; TFBUT, tear film breakup time; CFS, corneal fluorescein staining; MBI, maximum blink interval; iOS, iPhone operating system.

### Internal consistency and agreement of app-based MBI

Table 2 shows the internal consistency of app-based MBIs, with Cronbach’s alpha coefficients and intraclass correlation coefficients (ICCs). The Cronbach’s alpha coefficients of the correlation between app-based and visually confirmed MBIs were above 0.7 in the iOS (0.999) and Android (1.000) versions. The ICCs (95% confidence intervals [CIs]) of the app-based (0.996 [0.994–0.998], iOS; and 0.998 [0.997–0.999], Android) and visually confirmed MBIs were above 0.7.

**Table 2:** Reliability of app-based MBIs using Cronbach’s alpha and intraclass correlation coefficient values.

MBIs	Cronbach’s alpha	ICC (95% CI)
	n = 83	n = 83
App-based MBI (iOS) vs visually confirmed MBI (iOS)	0.999	0.996 (0.994–0.998)
App-based MBI (Android) vs visually confirmed MBI (Android)	1.000	0.998 (0.997–0.999)
App-based MBI (iOS) vs slit-lamp-based MBI	0.850	0.732 (0.615–0.818)
App-based MBI (Android) vs slit-lamp-based MBI	0.849	0.743 (0.630–0.826)
Visually confirmed MBI (iOS) vs slit-lamp-based MBI	0.849	0.735 (0.618–0.820)
Visually confirmed MBI (Android) vs slit-lamp-based MBI	0.852	0.744 (0.631–0.827)

ICC, intraclass correlation coefficient; MBI, maximum blink interval; CI, confidence interval; iOS, iPhone Operating System.

### Discriminant validity of app-based and slit-lamp-based MBIs

Table 3 shows the discriminant validity of app-based and slit-lamp-based MBIs. All MBIs were significantly shorter in the DED than in the non-DED groups (app-based MBI [iOS], *P* = 0.021; visually confirmed MBI [iOS], *P* = 0.018; app-based MBI [Android], *P* = 0.028; and visually confirmed MBI [Android], *P* = 0.031).

**Table 3:** Discriminant validity of app-based and visually confirmed MBIs.

MBIs	Non-DED	DED	*P* value	Total
	n = 32	n = 51		n = 83
App-based MBI (iOS), s ± SD	12.2 ± 7.9	8.3 ± 5.3	0.021	9.8 ± 6.7
Visually confirmed MBI (iOS), s ± SD	12.3 ± 7.9	8.4 ± 5.2	0.018	9.9 ± 6.6
App-based MBI (Android), s ± SD	12.3 ± 7.3	8.9 ± 5.3	0.028	10.2 ± 6.3
Visually confirmed MBI (Android), s ± SD	12.3 ± 7.3	9.0 ± 6.3	0.031	10.3 ± 6.3

*P* values were determined using two-tailed Student’s t test for continuous variables and χ^2^ test for categorical variables. DED, dry eye disease; SD, standard deviation; MBI, maximum blink interval; iOS, iPhone operating system.

### Concurrent validity among app-based, visually confirmed, and slit-lamp-based MBIs

The concurrent validity among the app-based, visually confirmed, and slit-lamp-based MBIs was assessed using Pearson’s correlation analysis (Table 4). Significant positive correlations were identified between the app-based (iOS) and visually confirmed MBIs (iOS) (*r* = 0.999, *P* < 0.001), app-based (Android) and visually confirmed MBIs (Android) (*r* = 0.999, *P* < 0.001), and app-based (iOS) and app-based MBIs (Android) (*r* = 0.824, *P* < 0.001).

**Table 4:** Correlations among app-based, visually confirmed, and slit-lamp-based MBIs.

MBIs	App-based MBI (iOS)	Visually confirmed MBI (iOS)	App-based MBI (Android)	Visually confirmed MBI (Android)	Slit-lamp-based MBI
App-based MBI (iOS)	1.000				
Visually confirmed MBI (iOS)	0.999	1.000			
App-based MBI (Android)	0.824	0.821	1.000		
Visually confirmed MBI (Android)	0.830	0.827	0.999	1.000	
Slit-lamp-based MBI	0.739	0.738	0.738	0.744	1.000

Correlation coefficients were determined using Pearson’s correlation analysis. MBI, maximum blink interval; iOS, iPhone operating system.

### Bland–Altman analysis

**Figure 2:**
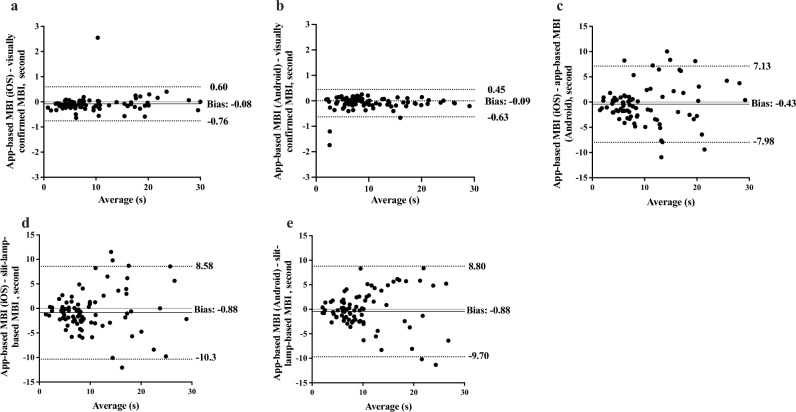
Bland–Altman plot for the app-based and visually confirmed maximum blink interval (MBI). The x-axis of the Bland–Altman plot represents average MBI values, and the y-axis represents differences between two of the different MBI measurement methods. The central line indicates the mean difference (bias) between the MBI values, whereas the superior and inferior lines indicate the upper and lower 95% limits of agreement, respectively. Differences between app-based and visually confirmed MBIs are displayed for (**a**) iOS and (**b**) Android. Differences in app-based MBI based on the operating systems are shown in (**c**). Differences between app-based and slit-lamp-based MBIs are displayed for (**d**) iOS and (**e**) Android. MBI, maximum blink interval; iOS, iPhone Operating System.

Bland–Altman analysis for agreement showed differences (biases) of − 0.08 s (Fig. 2a; 95% limits of agreement [LoA], − 0.76 to 0.60) between app-based (iOS) and visually confirmed MBIs (iOS), − 0.09 s (Fig. 2b; 95% LoA, − 0.63 to 0.45) between app-based (Android) and visually confirmed MBIs (Android), and -0.43 s (Fig. 2c; 95% LoA, − 7.98 to 7.13) between app-based (iOS) and app-based MBIs (Android) [− 0.88 s (Fig. 2d**;** 95% LoA, − 10.3 to 8.58) and -0.88 s (Fig. 2e; 95% LoA, − 9.70 to 8.80), respectively].

### Sensitivity, specificity, and cut-off values of app-based MBIs for detecting DED and TFBUT ≤ 5 s

Table 5 shows the sensitivity, specificity, and cut-off values for detecting DED determined by app-based and slit-lamp-based MBIs. For app-based MBI (iOS), the sensitivity, specificity, and area under the curve (AUC) for detecting DED were 80.4%, 50.0%, and 0.651, respectively, with an optimum cut-off value of 10.5 s. For the app-based MBI (Android), the sensitivity, specificity, and AUC for detecting DED were 49.0%, 78.1%, and 0.644, respectively, with an optimum cut-off value of 7.0 s. For slit-lamp-based MBI, the sensitivity, specificity, and AUC for detecting DED were 80.4%, 43.8%, and 0.638, respectively, with an optimum cut-off value of 11.7 s. Supplementary Tables S2–S4 show the cut-off values, sensitivity, specificity, and Youden indices of the app-based (iOS), app-based (Android), and slit-lamp-based MBIs for detecting DED.

**Table 5:** Sensitivity, specificity, cut-off value, and AUC for detecting DED.

MBIs	Sensitivity (%)	Specificity (%)	AUC	Optimum cut-off value (s)
App-based MBI (iOS)	80.4	50.0	0.651	10.5
App-based MBI (Android)	49.0	78.1	0.644	7.0
Slit-lamp-based MBI	80.4	43.8	0.638	11.7

AUC, area under the curve; DED, dry eye disease; MBI, maximum blink interval; iOS, iPhone operating system.

Table 6 shows the sensitivity, specificity, and cut-off values for detecting a TFBUT of ≤ 5 s determined by app-based and slit-lamp-based MBIs. For the app-based MBI (iOS), the sensitivity, specificity, and AUC for detecting DED were 44.3%, 76.9%, and 0.519, respectively, with an optimum cut-off value of 6.8 s. For the app-based MBI (Android), the sensitivity, specificity, and AUC for detecting DED were 85.7%, 30.7%, and 0.543, respectively, with an optimum cut-off value of 16.8 s. For the slit-lamp-based MBI, the sensitivity, specificity, and AUC for detecting DED were 72.9%, 38.5%, and 0.540, respectively, with an optimum cut-off value of 11.7 s. Supplementary Tables S5–S7 show the cut-off values, sensitivity, specificity, and Youden indices of the app-based (iOS), app-based (Android), and slit-lamp-based MBIs for detecting a TFBUT of ≤ 5 s.

**Table 6:** Sensitivity, specificity, cut-off value, and AUC for detecting TFBUT of ≤ 5 s.

MBIs	Sensitivity (%)	Specificity (%)	AUC	Optimum cut-off value (s)
App-based MBI (iOS)	44.3	76.9	0.519	6.8
App-based MBI (Android)	85.7	30.7	0.543	16.8
Slit-lamp-based MBI	72.9	38.5	0.540	11.7

AUC, area under the curve; TFBUT, tear film breakup time; MBI, maximum blink interval; iOS, iPhone operating system.

## Discussion

The unmet medical need for non-invasive, non-contact DED evaluation has drastically increased with the increase in DED prevalence due to aging and a digitalized society, as well as the COVID-19 pandemic. In this study, the performance of app-based MBI collected through the DryEyeRhythm app was compared with that of the slit-lamp-based MBI to determine its validity and reliability compared to the traditional technique. The results reflected the feasibility of the DryEyeRhythm app-based MBI in DED diagnosis and may help lay the foundation to implement digital phenotyping strategies and biometric data collection through mobile health (mHealth) apps. With a smartphone app for DED screening and management, DED care may become possible in a longitudinal, day-to-day manner with minimal invasiveness and no requirement to visit a specialized facility.

The synergy between mHealth and biometric data collection has been gaining attention owing to its potential for creating a comprehensive dataset on patient pathophysiology and elucidating new digital phenotypes with minimal intrusion^18–20^. Additionally, the push toward telemedicine has expanded substantially owing to the requirement of non-contact and non-invasive examinations during the COVID-19 pandemic^9^. However, robust evidence to support the implementation of mHealth- and app-based biometrics for clinical use is lacking^21^. In this study, we evaluated MBI biosensing using an image recognition app programming interface as part of a smartphone app to assist in DED diagnosis. Numerous approaches in incorporating mHealth and biosensing techniques have been used in ophthalmology, including visual acuity testing^22^, allergic conjunctivitis management^23^, pupillary reflex testing for amblyopia and strabismus detection^24^, and leukocoria recognition apps^25^. Their use is expected to expand with the rapidly increasing capabilities of commonplace smart devices and attached sensors. The unique advantage of mHealth can be attributed to its alignment with the paradigm shift from traditional facility-oriented medicine to non-intrusive, longitudinal care in a patient-centered manner^18^.

The results of this study demonstrate the validity, reliability, and equivalence of app-based MBI determination to its visually confirmed and slit-lamp-based counterparts. Good reliability values using Cronbach’s alpha coefficient and ICC were shown by both app-based and visually confirmed MBIs for iOS and Android platforms, reflecting sufficient internal consistency. App-based MBI also showed satisfactory discriminant validity and concurrent validity. Minimal biases were present between visually confirmed and app-based MBIs for both operating systems on Bland–Altman analysis. The discrepancy between the iOS and Android MBI measurements was minimal. Notably, the AUC of the app-based MBI for detecting decreased TFBUT was 0.519 and 0.543 for iOS and Android, respectively, possibly due to the temporal gap between app-based MBI and TFBUT measurements, which may be sufficient to affect the consistency of measurements. The demonstrated equivalence of app-based MBI with manually measured MBI and its reliability and validity suggest that app-based MBI measurements may be useful in obtaining an objective finding to support the diagnosis of DED in a telemedicine setting.

Two major criteria must be met to confirm a diagnosis of DED: subjective symptoms and objective clinical findings^6,12^. The Asia Dry Eye Society characterizes the pathophysiology of DED as a disease of tear film instability, which leads to visual decline^12^. TFBUT is a crucial component in assessing the tear film status^26^, and subjective symptoms quantified through disease-specific questionnaires alone are insufficient to make the diagnosis. However, specialized equipment and procedures (i.e., slit-lamp microscopy and fluorescein dye administration) are required to obtain TFBUT measurements, thus hindering remote assessment of DED status. Our previous efforts to find an appropriate substitute for TFBUT posited MBI as a possible candidate based on the positive correlation between the two measurements^15^. Additionally, the diagnostic performance of concomitant Japanese version of Ocular Surface Disease Index (J-OSDI) and MBI was satisfactory, with a sensitivity, specificity, and AUC of 75.4%, 92.9%, and 0.938, respectively^16^. The validity and reliability of the app-based J-OSDI were satisfactory, and its performance was comparable with its paper-based counterpart^5,14^. Therefore, accurate measurement of MBI using a smartphone app can enable comprehensive assessment of tear film status in a remote setting for DED diagnosis and progression monitoring. The results of this study demonstrate the validity, reliability, and equivalence of app-based MBI compared with the traditional measurement methods. Assisting DED diagnosis in a telemedicine environment may be possible by administering J-OSDI and measuring MBI using a smartphone app.

Our results indicate that the optimal cutoff values for app-based MBI were shorter, compared with the visually confirmed MBI. This discrepancy in MBI cutoff likely stems from the difference in visual tasks during various MBI measurements, with strong evidence supporting a significant decrease in blink rate and amplitude when using electronic monitors, such as smartphones and computers^27^. The observed decrease in optimal MBI cutoff for app-based measurements, compared with visually confirmed measurements, is thought to be affected by the specific visual task of focusing on handheld screens, which may ultimately elongate the physiological blink interval and subsequently the MBI when obtaining an app-based measurement. MBI cutoff values from prior studies were entirely derived under slit lamp-based measurements^15,16^. To enhance the assessment of different diagnostic capabilities of MBI and encourage its utilization on mobile platforms, future research should employ methodologies that effectively explore the optimal cutoff value for app-based MBI as a primary outcome^10^.

Previous efforts to screen for DED using web-based administered questionnaires lacked the objective component of DED diagnosis, such as TFBUT^28,29^. One notable strategy was to utilize an external infrared thermography device for smartphones, which showed satisfactory sensitivity, specificity, and AUC values of 96%, 91%, and 0.79, respectively^30^. However, specialized external devices are not ideal for screening purposes. DryEyeRhythm is an easily accessible software that can be executed by most commonplace smartphones to assess DED intermittently and longitudinally without the use of special devices or intrusive procedures. By administering the J-OSDI and measuring MBI through a single mHealth app, DryEyeRhythm, a comprehensive assessment of DED using remotely collected subjective and objective data on a patient’s tear film status may be possible.

This study has few limitations. First, it may have been affected by selection bias owing to its single-center design. The average TFBUT of the non-DED cohort participating in this study was 4.4 ± 2.5 s, lower than normal (range of normal TFBUT values: 7.6 ± 10.4 s to 9.1 ± 3.5 s)^11,12,31,32^. This may likely indicate that specialized university facilities are frequented by patients with various underlying ocular diseases that may affect tear film stability, and our sample may have included participants who may not accurately represent the larger population. Therefore, an ongoing multicenter, open-label, prospective, cross-sectional study is underway to determine the diagnostic ability of the smartphone app for DED and a cutoff value for app-based MBI^10^. Second, this study did not evaluate several factors associated with DED, such as socioeconomic status, education level, cultural background, lifestyle patterns, and systemic medications^1^. Third, as this study aimed to assess the reliability, validity, and equivalence of app-based MBI compared with traditional measurements, several objective findings were not evaluated, including Rose Bengal staining scores, tear osmolality, meibomian gland dysfunction, and corneal sensations. Lastly, this study excluded participants with ptosis or other palpebral dysfunctions that may physically disrupt normal blinking physiology. Therefore, the app may not accurately measure MBI in older patients with dermatochalasis. Additionally, as the blink recognition function of DryEyeRhythm can be hindered for users wearing a mask, the app-based MBI was measured with masks removed. Another factor that may affect the blink recognition function of DryEyeRhythm is the narrow palpebral fissure width of the participants^33^, due to which, 10 participants were unable to undergo app-based MBI measurements in this study. Future studies and updates of the app should focus on enhancing the recognition algorithm, aiming to eliminate the necessity for users to remove masks and adjusting for narrow palpebral fissure width.

In summary, MBI measured through DryEyeRhythm, an app available on iOS and Android platforms, showed good reliability, validity, and equivalence compared with slit-lamp-based MBI measurements, suggesting that app-based MBI could be a substitute for TFBUT in an mHealth environment. The results of this study indicate that DryEyeRhythm may serve as a novel tool for assisting in DED diagnosis and progression monitoring in a remote setting.

## Methods

### DryEyeRhythm smartphone application

The DryEyeRhythm smartphone app was initially developed using the open-source framework ResearchKit of Apple Inc. (Cupertino, CA, USA)^17^. The app was released in November 2016 for iOS and September 2020 for Android under a consignment contract with the Juntendo University Graduate School of Medicine, Tokyo, Japan, and InnoJin Inc., Tokyo, Japan. It is freely available on Apple’s App Store and Google Play.

The DryEyeRhythm app collects data regarding user demographics, medical history, lifestyle history, daily subjective symptoms, J-OSDI score, blink monitoring including blink frequency and MBI biosensor data, depression data (Zung Self-Rating Depression Scale), and work productivity^4,5,8,17,18,34–36^.

### Study design and participants

This single-center, prospective, cross-sectional, observational study was conducted at the Juntendo University Hospital, Department of Ophthalmology, Tokyo, Japan^37^. Patients aged ≥ 20 years were recruited between February 16, 2022, and August 3, 2022. Written informed consent was obtained from all participants. This study was approved by the Independent Ethics Committee of the Juntendo University Faculty of Medicine (approval number: 20-092) in accordance with the Declaration of Helsinki.

Participants with a history of eyelid disorders, ptosis, psychiatric disease, Parkinson’s disease, or any other disease affecting blinking were excluded^5,15^. Those with any missing data and whose MBI measurements could not be obtained with the DryEyeRhythm app were also excluded.

### Dry eye disease diagnosis

According to the 2016 Asia Dry Eye Society criteria^12^, participants with a TFBUT ≤ 5 s and J-OSDI ≥ 13 points were diagnosed with DED. The TFBUT was considered positive if the average was ≤ 5 s in a severely affected eye.

### MBI

**Figure 3:**
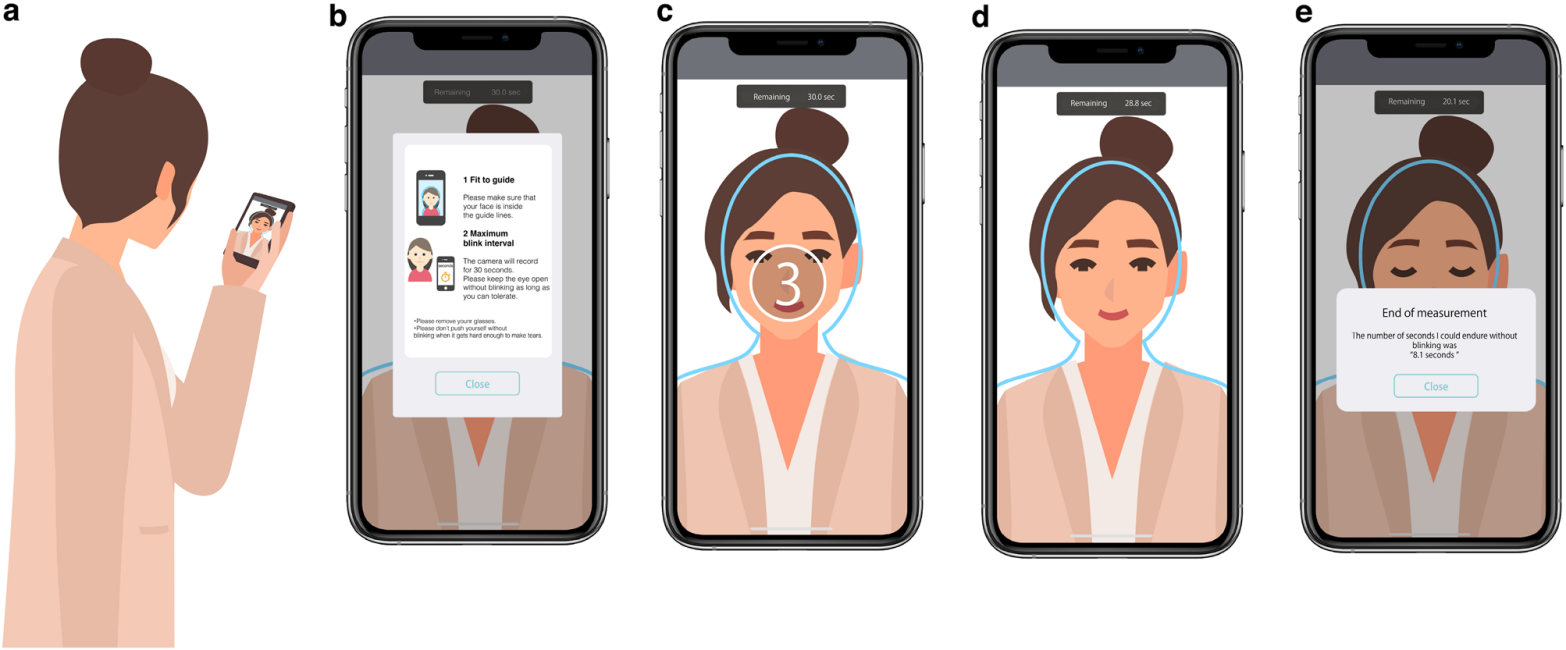
Illustration showing the process of DryEyeRhythm application-based maximum blink interval (MBI) measurement. (**a**) Representative illustration of MBI measurement using the DryEyeRhythm app. (**b**) Prior to the app-based MBI measurement, instructional information on the procedure is displayed to the user. (**c**) The user must align their face with the displayed guideline during the 3-s countdown before MBI measurement. (**d**) MBI measurement screen: measurements are obtained with the face aligned to the guideline. (**e**) Measurement completion screen: once the app detects a blink, the MBI is displayed to the user and the measurement is complete.

MBI was defined as the time that patients could keep their eyes open before blinking^15^. It was measured in three ways: using a slit-lamp microscope (slit-lamp-based MBI), DryEyeRhythm (app-based MBI [iOS and Android]), and a stopwatch (visually confirmed MBI). All MBIs were measured thrice. Slit-lamp-based MBI was calculated using a stopwatch under light microscopy. App-based MBIs were measured using the iOS and Android versions of the DryEyeRhythm smartphone app installed on an iPhone 12 Pro MAX (Apple Inc., Cupertino, CA, USA) and an Xperia 5 II (Sony Corporation, Tokyo, Japan) and their embedded cameras, with a face recognition technology called ARCore for the iOS and Android interface^38^. During the measurement of app-based MBIs, visually confirmed MBI was measured by the examiner by observing the user’s eyes with a stopwatch. The mean MBI was used in the analysis. Figure 3 shows a representative illustration (Fig. 3a) and screenshots of MBI measurement (Fig. 3b–e**)** using the DryEyeRhythm app.

### Study procedures

Figure 1 shows the flowchart of this study. All participants underwent visual acuity measurement; intraocular pressure measurement; and several DED evaluations, including TFBUT, corneal fluorescein staining (CFS), and slit-lamp-based MBI. After completing these tests, the app-based and visually confirmed MBIs were measured simultaneously. A Schirmer test I was performed after the MBI measurement.

### Clinical assessment of DED

TFBUT was measured using fluorescein sodium staining (Ayumi Pharmaceutical Co., Tokyo, Japan) according to standard methodology^12^. The mean values of three measurements in the right eye were used for the analysis. CFS was graded according to the van Bijsterveld grading score^39^, with a maximum potential score of 9. Schirmer’s test I was performed without topical anesthesia after completing all examinations.

### Reliability

The internal consistency of the app-based MBIs was assessed using Cronbach’s alpha coefficient; an alpha value > 0.70 was considered acceptable^40^. ICC was used to evaluate the agreement among the slit-lamp-based, app-based, and visually confirmed MBIs. An ICC value ≥ 0.70 was considered acceptable^41^.

### Validity

The discriminant validity of each MBI was evaluated by comparing the non-DED and DED groups. Concurrent validity was assessed by calculating correlations (Pearson coefficients) between each app-based MBI. Bland–Altman analysis^42^ was conducted to indicate systematic random error, heteroscedasticity of the data, and 95% LoA of each MBI.

### Statistical analysis

The sample size was predetermined using the formula derived from hypothesis testing^43^. Using the following settings, the required sample size was determined to be 79 cases: power, 80%; significance level, 5%; minimal acceptable ICC score, 0.5; expected ICC score, 0.7; and number of raters, 2. Considering a dropout rate of 15% possibly owing to missing data, unmeasurable app-based MBIs, and withdrawal of consent, 94 participants were recruited.

The characteristics of study participants were compared using an unpaired t test for continuous variables and an χ^2^ test for categorical variables. Data were presented as mean ± standard deviation or percentage. Receiver operating characteristic curve analysis was performed to examine the diagnostic efficacy of MBIs in detecting DED or TFBUT ≤ 5 s. The AUC was estimated using the trapezoidal rule^4^. The cut-off values of MBIs for detecting DED and TFBUT ≤ 5 s were determined using the Youden index^44^. Statistical analyses were performed using STATA version 15 (StataCorp, College Station, TX, USA), and statistical significance was set at *P* < 0.05.

### Supplementary Information

Supplementary Tables.

## Data Availability

All data are available in the main text or the supplementary materials. Data access, responsibility, and analysis: Takenori Inomata, had full access to all the data in the study and takes responsibility for the integrity of the data and the accuracy of data analysis.

## References

[1] Stapleton F (2017). TFOS DEWS II epidemiology report. Ocul. Surf..

[2] Inomata T (2018). Changes in distribution of dry eye disease by the new 2016 diagnostic criteria from the Asia dry eye society. Sci. Rep..

[3] Wolffsohn JS (2023). TFOS lifestyle: Impact of the digital environment on the ocular surface. Ocul. Surf..

[4] Midorikawa-Inomata A (2019). Reliability and validity of the Japanese version of the Ocular Surface Disease Index for dry eye disease. BMJ Open.

[5] Okumura Y (2022). DryEyeRhythm: A reliable and valid smartphone application for the diagnosis assistance of dry eye. Ocul. Surf..

[6] Craig JP (2017). TFOS DEWS II definition and classification report. Ocul. Surf..

[7] Yamada M, Mizuno Y, Shigeyasu C (2012). Impact of dry eye on work productivity. Clinicoecon. Outcomes Res..

[8] Inomata T (2020). Characteristics and risk factors associated with diagnosed and undiagnosed symptomatic dry eye using a smartphone application. JAMA Ophthalmol..

[9] Kim S, Kim JA, Lee JY (2022). International trend of non-contact healthcare and related changes due to COVID-19 pandemic. Yonsei Med. J..

[10] Nagino K (2023). Diagnostic ability of a smartphone app for dry eye disease: protocol for a multicenter, open-label, prospective, and cross-sectional study. JMIR Res. Protoc..

[11] Wolffsohn JS (2017). TFOS DEWS II diagnostic methodology report. Ocul. Surf..

[12] Tsubota K (2017). New perspectives on dry eye definition and diagnosis: A consensus report by the Asia dry eye society. Ocul. Surf..

[13] Inomata T (2020). Using medical big data to develop personalized medicine for dry eye disease. Cornea.

[14] Nagino K (2023). Smartphone app-based and paper-based patient-reported outcomes using a disease-specific questionnaire for dry eye disease: randomized crossover equivalence study. J. Med. Internet. Res..

[15] Inomata T (2018). Maximum blink interval is associated with tear film breakup time: A new simple, screening test for dry eye disease. Sci. Rep..

[16] Hirosawa K (2020). Diagnostic ability of maximum blink interval together with Japanese version of Ocular Surface Disease Index score for dry eye disease. Sci. Rep..

[17] Inomata T (2019). Risk factors for severe dry eye disease: Crowdsourced research using DryEyeRhythm. Ophthalmology.

[18] Inomata T (2021). Smartphone-based digital phenotyping for dry eye toward P4 medicine: A crowdsourced cross-sectional study. NPJ. Digit. Med..

[19] Yang Q (2020). Digital phenotyping self-monitoring behaviors for individuals with type 2 diabetes mellitus: Observational study using latent class growth analysis. JMIR Mhealth Uhealth.

[20] Nagino K (2023). Symptom-based stratification algorithm for heterogeneous symptoms of dry eye disease: A feasibility study. Eye.

[21] Milne-Ives M, Lam C, De Cock C, Van Velthoven MH, Meinert E (2020). Mobile apps for health behavior change in physical activity, diet, drug and alcohol use, and mental health: Systematic review. JMIR Mhealth Uhealth.

[22] Bastawrous A (2015). Development and validation of a smartphone-based visual acuity test (peek acuity) for clinical practice and Community-Based Fieldwork. JAMA Ophthalmol..

[23] Inomata T (2022). Individual characteristics and associated factors of hay fever: A large-scale mHealth study using AllerSearch. Allergol. Int..

[24] Arnold RW, O'Neil JW, Cooper KL, Silbert DI, Donahue SP (2018). Evaluation of a smartphone photoscreening app to detect refractive amblyopia risk factors in children aged 1–6 years. Clin. Ophthalmol..

[25] Vagge A (2019). Evaluation of a free public smartphone application to detect Leukocoria in high-risk children aged 1 to 6 years. J. Pediatr. Ophthalmol. Strabismus.

[26] Yokoi N, Georgiev GA (2018). Tear film-oriented diagnosis and tear film-oriented therapy for dry eye based on tear film dynamics. Invest. Ophthalmol. Vis. Sci..

[27] Jaiswal S (2019). Ocular and visual discomfort associated with smartphones, tablets and computers: What we do and do not know. Clin. Exp. Optom..

[28] Kawashima M (2013). A field test of Web-based screening for dry eye disease to enhance awareness of eye problems among general Internet users: A latent strategy to promote health. J. Med. Internet Res..

[29] Amparo F, Dana R (2018). Web-based longitudinal remote assessment of dry eye symptoms. Ocul. Surf..

[30] Zhang Q (2021). Screening evaporative dry eyes severity using an infrared image. J. Ophthalmol..

[31] Singh S, Srivastav S, Modiwala Z, Ali MH, Basu S (2023). Repeatability, reproducibility and agreement between three different diagnostic imaging platforms for tear film evaluation of normal and dry eye disease. Eye.

[32] Nichols JJ, Nichols KK, Puent B, Saracino M, Mitchell GL (2002). Evaluation of tear film interference patterns and measures of tear break-up time. Optom. Vis. Sci..

[33] Chen S, Epps J (2019). Eyelid and pupil landmark detection and blink estimation based on deformable shape models for near-field infrared video. Front. ICT.

[34] Inomata T (2020). Association between dry eye and depressive symptoms: Large-scale crowdsourced research using the DryEyeRhythm iPhone application. Ocul. Surf..

[35] Inomata T (2020). Stratification of individual symptoms of contact lens-associated dry eye using the iPhone App DryEyeRhythm: Crowdsourced cross-sectional study. J. Med. Internet Res..

[36] Eguchi A (2021). Heterogeneity of eye drop use among symptomatic dry eye individuals in Japan: Large-scale crowdsourced research using DryEyeRhythm application. Jpn. J. Ophthalmol..

[37] Inomata T (2018). The impact of Joint Commission International accreditation on time periods in the operating room: A retrospective observational study. PLoS ONE.

[38] GoogleInc. *ARCore*, <https://developers.google.com/ar>

[39] van Bijsterveld OP (1969). Diagnostic tests in the Sicca syndrome. Arch. Ophthalmol..

[40] Cronbach LJ (1951). Coefficient alpha and the internal structure of tests. Psychometrika.

[41] Deyo RA, Diehr P, Patrick DL (1991). Reproducibility and responsiveness of health status measures. Statistics and strategies for evaluation. Control. Clin. Trials.

[42] Bland JM, Altman DG (1986). Statistical methods for assessing agreement between two methods of clinical measurement. Lancet.

[43] Walter SD, Eliasziw M, Donner A (1998). Sample size and optimal designs for reliability studies. Stat. Med..

[44] Fluss R, Faraggi D, Reiser B (2005). Estimation of the Youden Index and its associated cutoff point. Biom. J..

