# Supplementary material for: Clinical utility of maximum blink interval measured by smartphone application DryEyeRhythm to support dry eye disease diagnosis
Source: Sci Rep. 2023 Aug 21;13:13583. doi: 10.1038/s41598-023-40968-y (PMC10442434; doi:10.1038/s41598-023-40968-y)
Supplement: Supplementary file 1 — Supplementary Tables. [file 41598_2023_40968_MOESM1_ESM.pdf]

Supplementary material

Supplementary Table S1. Comparison between included and excluded participants

**Characteristics**	**Excluded n=10**	**Included n=83**	***P* Value**
Age, year ± SD	76.1±7.0	62.0±13.1	< 0.001
Sex, female (%)	8 (80.0)	78 (94.0)	0.114
J-OSDI, 0–100 ± SD	28.2±22.1	29.1±20.6	0.941
TFBUT, s ± SD	3.1±1.7	3.4±2.0	0.901
CFS, 0–9 ± SD	1.5±1.4	2.9±2.5	0.078
Schirmer I, mm ± SD	6.4±9.2	7.7±7.7	0.311
Slit-lamp-based MBI, s ± SD	11.8±4.7	10.7±7.0	0.206

SD, standard deviation; J-OSDI, Japanese version of the Ocular Surface Disease Index; TFBUT, tear film breakup time; CFS, corneal fluorescence staining; MBI, maximum blink interval

Supplementary Table S2. Sensitivity and specificity of the app-based MBI (iOS) for the diagnosis of dry eye disease

Cutoff value of app-based MBI (iOS) (s)	Sensitivity (%)	Specificity (%)	Youden index
< 0.9333	0	96.88	-0.031
< 1.717	1.961	96.88	-0.012
< 2.417	3.922	93.75	-0.023
< 2.750	5.882	93.75	-0.004
< 2.950	7.843	93.75	0.016
< 3.233	9.804	93.75	0.036
< 3.550	11.76	93.75	0.055
< 3.717	11.76	90.63	0.024
< 3.917	11.76	87.5	-0.007
< 4.067	11.76	84.38	-0.039
< 4.150	13.73	81.25	-0.050
< 4.250	15.69	81.25	-0.031
< 4.483	17.65	81.25	-0.011
< 4.717	19.61	81.25	0.009
< 4.817	21.57	81.25	0.028
< 4.883	23.53	81.25	0.048
< 4.950	25.49	81.25	0.067
< 5.050	27.45	81.25	0.087
< 5.200	29.41	81.25	0.107
< 5.383	31.37	81.25	0.126
< 5.517	31.37	78.13	0.095
< 5.650	33.33	78.13	0.115
< 5.750	35.29	78.13	0.134
< 5.783	37.25	78.13	0.154
< 5.833	39.22	78.13	0.174
< 5.883	45.10	78.13	0.232

< 5.917	47.06	78.13	0.252
< 6.017	49.02	78.13	0.272
< 6.433	49.02	75.00	0.240
< 6.817	50.98	75.00	0.260
< 6.900	52.94	71.88	0.248
< 6.983	52.94	68.75	0.217
< 7.067	54.90	68.75	0.237
< 7.200	56.86	68.75	0.256
< 7.317	58.82	68.75	0.276
< 7.383	58.82	65.63	0.245
< 7.483	60.78	65.63	0.264
< 7.550	62.75	65.63	0.284
< 7.617	62.75	59.38	0.221
< 7.700	64.71	59.38	0.241
< 7.750	66.67	59.38	0.261
< 7.883	68.63	59.38	0.280
< 8.383	70.59	59.38	0.300
< 9.067	70.59	56.25	0.268
< 9.400	70.59	53.13	0.237
< 9.650	70.59	50.00	0.206
< 10.05	72.55	50.00	0.226
< 10.27	74.51	50.00	0.245
< 10.33	76.47	50.00	0.265
< 10.38	78.43	50.00	0.284
< 10.48	80.39	50.00	0.304
< 10.58	80.39	46.88	0.273
< 10.63	80.39	43.75	0.241
< 11.13	80.39	40.63	0.210
< 12.02	82.35	40.63	0.230
< 13.18	82.35	37.50	0.199
< 14.55	84.31	37.50	0.218
< 15.28	86.27	37.50	0.238

< 15.68	86.27	34.38	0.207
< 16.30	86.27	31.25	0.175
< 16.68	88.24	31.25	0.195
< 17.03	88.24	28.13	0.164
< 17.52	90.20	28.13	0.183
< 17.72	92.16	28.13	0.203
< 18.02	94.12	28.13	0.223
< 18.45	94.12	25.00	0.191
< 18.83	94.12	21.88	0.160
< 19.18	94.12	18.75	0.129
< 19.58	94.12	15.63	0.098
< 19.93	94.12	12.50	0.066
< 20.18	96.08	12.50	0.086
< 21.13	96.08	9.375	0.055
< 22.82	98.04	9.375	0.074
< 25.78	100	9.375	0.094
< 28.62	100	6.25	0.063
< 29.70	100	3.125	0.031

App, application; MBI, maximum blink interval; iOS, iPhone operating system

Supplementary Table S3. Sensitivity and specificity of the app-based MBI (Android) for the diagnosis of dry eye disease

Cut-off value of app-based MBI (Android) (s)	Sensitivity (%)	Specificity (%)	Youden index
< 1.833	1.961	100	0.020
< 1.983	3.922	100	0.039
< 2.133	5.882	100	0.059
< 2.350	5.882	96.88	0.028
< 2.467	7.843	96.88	0.047
< 2.900	9.804	96.88	0.067
< 3.533	9.804	93.75	0.036
< 3.800	11.76	93.75	0.055
< 3.900	13.73	93.75	0.075
< 4.000	13.73	90.63	0.044
< 4.233	15.69	90.63	0.063
< 4.683	17.65	90.63	0.083
< 4.942	19.61	90.63	0.102
< 5.008	21.57	90.63	0.122
< 5.150	23.53	90.63	0.142
< 5.317	23.53	87.50	0.110
< 5.433	23.53	84.38	0.079
< 5.600	25.49	84.38	0.099
< 5.783	27.45	84.38	0.118
< 5.867	27.45	81.25	0.087
< 5.917	29.41	81.25	0.107
< 6.033	31.37	81.25	0.126
< 6.150	33.33	81.25	0.146
< 6.233	35.29	81.25	0.165
< 6.400	37.25	81.25	0.185
< 6.583	39.22	81.25	0.205

< 6.750	43.14	81.25	0.244
< 6.842	43.14	78.13	0.213
< 6.892	45.10	78.13	0.232
< 6.950	47.06	78.13	0.252
< 7.000	49.02	78.13	0.272
< 7.083	50.98	75.00	0.260
< 7.283	50.98	71.88	0.229
< 7.450	52.94	71.88	0.248
< 7.500	52.94	68.75	0.217
< 7.717	54.90	68.75	0.237
< 7.967	56.86	68.75	0.256
< 8.117	56.86	65.63	0.225
< 8.217	56.86	62.50	0.194
< 8.267	56.86	59.38	0.162
< 8.467	58.82	59.38	0.182
< 8.717	58.82	56.25	0.151
< 8.875	60.78	53.13	0.139
< 9.075	62.75	53.13	0.159
< 9.233	64.71	53.13	0.178
< 9.383	64.71	50.00	0.147
< 9.650	64.71	46.88	0.116
< 9.867	64.71	43.75	0.085
< 9.950	66.67	43.75	0.104
< 10.07	68.63	43.75	0.124
< 10.38	70.59	43.75	0.143
< 10.65	72.55	43.75	0.163
< 11.02	72.55	40.63	0.132
< 11.67	74.51	40.63	0.151
< 12.30	76.47	40.63	0.171
< 12.75	78.43	40.63	0.191
< 13.18	80.39	40.63	0.210
< 13.58	80.39	37.50	0.179

< 13.77	82.35	37.50	0.199
< 13.98	84.31	37.50	0.218
< 14.30	84.31	34.38	0.187
< 14.77	86.27	34.38	0.207
< 15.30	86.27	31.25	0.175
< 15.58	88.24	31.25	0.195
< 16.07	90.20	31.25	0.215
< 16.75	90.20	28.13	0.183
< 17.18	90.20	25.00	0.152
< 18.02	90.20	18.75	0.090
< 18.75	92.16	18.75	0.109
< 19.35	94.12	18.75	0.129
< 20.00	96.08	18.75	0.148
< 20.62	96.08	15.63	0.117
< 21.23	98.04	15.63	0.137
< 22.48	98.04	12.50	0.105
< 23.88	98.04	9.375	0.074
< 25.15	100	9.375	0.094
< 26.20	100	6.25	0.063
< 27.63	100	3.125	0.031

App, application; MBI, maximum blink interval

Supplementary Table S4. Sensitivity and specificity of the slit-lamp-based MBI for the diagnosis of dry eye disease

Cut-off value for slit-lamp-based MBI (s)	Sensitivity (%)	Specificity (%)	Youden index
< 1.900	0	96.88	-0.031
< 2.183	0	93.75	-0.063
< 2.533	1.961	93.75	-0.043
< 2.683	3.922	93.75	-0.023
< 2.833	5.882	93.75	-0.004
< 3.050	7.843	93.75	0.016
< 3.783	9.804	93.75	0.036
< 4.467	11.76	93.75	0.055
< 4.633	11.76	90.63	0.024
< 4.883	15.69	90.63	0.063
< 5.083	17.65	90.63	0.083
< 5.150	21.57	90.63	0.122
< 5.283	23.53	87.50	0.110
< 5.500	25.49	87.50	0.130
< 5.617	27.45	84.38	0.118
< 5.667	29.41	84.38	0.138
< 5.850	31.37	84.38	0.158
< 6.117	33.33	84.38	0.177
< 6.367	33.33	81.25	0.146
< 6.567	33.33	78.13	0.115
< 6.733	35.29	78.13	0.134
< 6.883	35.29	75.00	0.103
< 7.067	37.25	75.00	0.123
< 7.250	39.22	75.00	0.142
< 7.350	39.22	71.88	0.111
< 7.433	41.18	71.88	0.131
< 7.500	43.14	71.88	0.150

< 7.667	45.10	71.88	0.170
< 7.883	45.10	68.75	0.139
< 7.983	47.06	68.75	0.158
< 8.150	49.02	68.75	0.178
< 8.317	50.98	68.75	0.197
< 8.367	50.98	65.63	0.166
< 8.417	52.94	65.63	0.186
< 8.442	52.94	62.50	0.154
< 8.725	54.90	62.50	0.174
< 9.117	56.86	62.50	0.194
< 9.250	58.82	62.50	0.213
< 9.300	60.78	59.38	0.202
< 9.400	62.75	59.38	0.221
< 9.483	64.71	59.38	0.241
< 9.533	64.71	56.25	0.210
< 9.583	66.67	56.25	0.229
< 9.667	66.67	53.13	0.198
< 9.750	68.63	53.13	0.218
< 9.933	70.59	53.13	0.237
< 10.12	72.55	53.13	0.257
< 10.23	74.51	53.13	0.276
< 10.45	76.47	53.13	0.296
< 10.63	76.47	50.00	0.265
< 11.02	78.43	50.00	0.284
< 11.38	80.39	50.00	0.304
< 11.53	80.39	46.88	0.273
< 11.68	80.39	43.75	0.241
< 12.10	82.35	43.75	0.261
< 12.83	82.35	40.63	0.230
< 13.25	84.31	40.63	0.249
< 13.50	86.27	40.63	0.269
< 13.95	88.24	40.63	0.289

< 14.65	88.24	34.38	0.226
< 15.22	88.24	31.25	0.195
< 15.48	88.24	28.13	0.164
< 16.70	88.24	25.00	0.132
< 17.78	88.24	21.88	0.101
< 18.07	88.24	18.75	0.070
< 18.88	90.2	18.75	0.090
< 20.25	90.2	15.63	0.058
< 21.25	90.2	12.5	0.027
< 21.87	90.2	9.375	-0.004
< 22.37	92.16	9.375	0.015
< 23.07	94.12	9.375	0.035
< 23.73	96.08	9.375	0.055
< 25.23	96.08	6.25	0.023
< 28.23	96.08	3.125	-0.008
< 29.88	98.04	3.125	0.012

MBI: maximum blink interval

Supplementary Table S5. Sensitivity and specificity of the app-based MBI (iOS) for tear-film breakup time

Cut-off value for app-based MBI (iOS) (s)	Sensitivity (%)	Specificity (%)	Youden index
< 0.9333	1.429	100	0.014
< 1.717	2.857	100	0.029
< 2.417	4.286	92.31	-0.034
< 2.750	5.714	92.31	-0.020
< 2.950	7.143	92.31	-0.005
< 3.233	8.571	92.31	0.009
< 3.550	10.00	92.31	0.023
< 3.717	10.00	84.62	-0.054
< 3.917	10.00	76.92	-0.131
< 4.067	11.43	76.92	-0.117
< 4.150	14.29	76.92	-0.088
< 4.250	15.71	76.92	-0.074
< 4.483	17.14	76.92	-0.059
< 4.717	18.57	76.92	-0.045
< 4.817	20.00	76.92	-0.031
< 4.883	21.43	76.92	-0.017
< 4.950	22.86	76.92	-0.002
< 5.050	24.29	76.92	0.012
< 5.200	25.71	76.92	0.026
< 5.383	27.14	76.92	0.041
< 5.517	28.57	76.92	0.055
< 5.650	30.00	76.92	0.069
< 5.750	31.43	76.92	0.083
< 5.783	32.86	76.92	0.098
< 5.833	34.29	76.92	0.112
< 5.883	38.57	76.92	0.155

< 5.917	40.00	76.92	0.169
< 6.017	41.43	76.92	0.184
< 6.433	42.86	76.92	0.198
< 6.817	44.29	76.92	0.212
< 6.900	45.71	69.23	0.149
< 6.983	45.71	61.54	0.073
< 7.067	47.14	61.54	0.087
< 7.200	48.57	61.54	0.101
< 7.317	50.00	61.54	0.115
< 7.383	51.43	61.54	0.130
< 7.483	52.86	61.54	0.144
< 7.550	54.29	61.54	0.158
< 7.617	55.71	53.85	0.096
< 7.700	57.14	53.85	0.110
< 7.750	58.57	53.85	0.124
< 7.883	60.00	53.85	0.139
< 8.383	61.43	53.85	0.153
< 9.067	61.43	46.15	0.076
< 9.400	61.43	38.46	-0.001
< 9.650	61.43	30.77	-0.078
< 10.05	62.86	30.77	-0.064
< 10.27	64.29	30.77	-0.049
< 10.33	65.71	30.77	-0.035
< 10.38	67.14	30.77	-0.021
< 10.48	68.57	30.77	-0.007
< 10.58	70.00	30.77	0.008
< 10.63	71.43	30.77	0.022
< 11.13	72.86	30.77	0.036
< 12.02	74.29	30.77	0.051
< 13.18	74.29	23.08	-0.026
< 14.55	75.71	23.08	-0.012
< 15.28	77.14	23.08	0.002

< 15.68	77.14	15.38	-0.075
< 16.30	78.57	15.38	-0.061
< 16.68	80.00	15.38	-0.046
< 17.03	80.00	7.692	-0.123
< 17.52	81.43	7.692	-0.109
< 17.72	82.86	7.692	-0.094
< 18.02	84.29	7.692	-0.080
< 18.45	85.71	7.692	-0.066
< 18.83	87.14	7.692	-0.052
< 19.18	88.57	7.692	-0.037
< 19.58	90.00	7.692	-0.023
< 19.93	90.00	0	-0.100
< 20.18	91.43	0	-0.086
< 21.13	92.86	0	-0.071
< 22.82	94.29	0	-0.057
< 25.78	95.71	0	-0.043
< 28.62	97.14	0	-0.029
< 29.70	98.57	0	-0.014

App, application; MBI, maximum blink interval; iOS, iPhone operating system

Supplementary Table S6. Sensitivity and specificity of the app-based MBI (Android) for tear-film breakup time

Cut-off value for app-based MBI (Android) (s)	Sensitivity (%)	Specificity (%)	Youden index
< 1.833	1.429	100	0.014
< 1.983	2.857	100	0.029
< 2.133	4.286	100	0.043
< 2.350	5.714	100	0.057
< 2.467	7.143	100	0.071
< 2.900	8.571	100	0.086
< 3.533	8.571	92.31	0.009
< 3.800	10.00	92.31	0.023
< 3.900	11.43	92.31	0.037
< 4.000	11.43	84.62	-0.039
< 4.233	12.86	84.62	-0.025
< 4.683	14.29	84.62	-0.011
< 4.942	15.71	84.62	0.003
< 5.008	17.14	84.62	0.018
< 5.150	18.57	84.62	0.032
< 5.317	20.00	84.62	0.046
< 5.433	20.00	76.92	-0.031
< 5.600	21.43	76.92	-0.017
< 5.783	22.86	76.92	-0.002
< 5.867	24.29	76.92	0.012
< 5.917	25.71	76.92	0.026
< 6.033	27.14	76.92	0.041
< 6.150	28.57	76.92	0.055
< 6.233	30.00	76.92	0.069
< 6.400	31.43	76.92	0.083
< 6.583	32.86	76.92	0.098

< 6.750	35.71	76.92	0.126
< 6.842	35.71	69.23	0.049
< 6.892	37.14	69.23	0.064
< 6.950	38.57	69.23	0.078
< 7.000	40.00	69.23	0.092
< 7.083	42.86	69.23	0.121
< 7.283	42.86	61.54	0.044
< 7.450	44.29	61.54	0.058
< 7.500	44.29	53.85	-0.019
< 7.717	45.71	53.85	-0.004
< 7.967	47.14	53.85	0.010
< 8.117	48.57	53.85	0.024
< 8.217	50.00	53.85	0.038
< 8.267	51.43	53.85	0.053
< 8.467	52.86	53.85	0.067
< 8.717	52.86	46.15	-0.010
< 8.875	55.71	46.15	0.019
< 9.075	57.14	46.15	0.033
< 9.233	58.57	46.15	0.047
< 9.383	60.00	46.15	0.062
< 9.650	61.43	46.15	0.076
< 9.867	61.43	38.46	-0.001
< 9.950	62.86	38.46	0.013
< 10.07	64.29	38.46	0.028
< 10.38	65.71	38.46	0.042
< 10.65	67.14	38.46	0.056
< 11.02	68.57	38.46	0.070
< 11.67	70.00	38.46	0.085
< 12.30	71.43	38.46	0.099
< 12.75	72.86	38.46	0.113
< 13.18	74.29	38.46	0.128
< 13.58	74.29	30.77	0.051

< 13.77	75.71	30.77	0.065
< 13.98	77.14	30.77	0.079
< 14.30	78.57	30.77	0.093
< 14.77	80.00	30.77	0.108
< 15.30	81.43	30.77	0.122
< 15.58	82.86	30.77	0.136
< 16.07	84.29	30.77	0.151
< 16.75	85.71	30.77	0.165
< 17.18	85.71	23.08	0.088
< 18.02	85.71	7.692	-0.066
< 18.75	87.14	7.692	-0.052
< 19.35	88.57	7.692	-0.037
< 20.00	90.00	7.692	-0.023
< 20.62	91.43	7.692	-0.009
< 21.23	92.86	7.692	0.006
< 22.48	94.29	7.692	0.020
< 23.88	95.71	7.692	0.034
< 25.15	97.14	7.692	0.048
< 26.20	97.14	0	-0.029
< 27.63	98.57	0	-0.014

MBI: maximum blink interval

Supplementary Table S7. Sensitivity and specificity of the slit-lamp-based MBI for tear-film breakup time

Cut-off value for slit-lamp-based MBI (s)	Sensitivity (%)	Specificity (%)	Youden index
< 1.900	1.429	100	0.014
< 2.183	1.429	92.31	-0.063
< 2.533	2.857	92.31	-0.048
< 2.683	4.286	92.31	-0.034
< 2.833	5.714	92.31	-0.020
< 3.050	7.143	92.31	-0.005
< 3.783	8.571	92.31	0.009
< 4.467	10.00	92.31	0.023
< 4.633	11.43	92.31	0.037
< 4.883	14.29	92.31	0.066
< 5.083	15.71	92.31	0.080
< 5.150	18.57	92.31	0.109
< 5.283	20.00	84.62	0.046
< 5.500	21.43	84.62	0.061
< 5.617	22.86	76.92	-0.002
< 5.667	24.29	76.92	0.012
< 5.850	25.71	76.92	0.026
< 6.117	27.14	76.92	0.041
< 6.367	28.57	76.92	0.055
< 6.567	30.00	76.92	0.069
< 6.733	31.43	76.92	0.083
< 6.883	31.43	69.23	0.007
< 7.067	32.86	69.23	0.021
< 7.250	34.29	69.23	0.035
< 7.350	34.29	61.54	-0.042
< 7.433	35.71	61.54	-0.028
< 7.500	37.14	61.54	-0.013

< 7.667	38.57	61.54	0.001
< 7.883	40.00	61.54	0.015
< 7.983	41.43	61.54	0.030
< 8.150	42.86	61.54	0.044
< 8.317	44.29	61.54	0.058
< 8.367	44.29	53.85	-0.019
< 8.417	45.71	53.85	-0.004
< 8.442	47.14	53.85	0.010
< 8.725	48.57	53.85	0.024
< 9.117	50.00	53.85	0.038
< 9.250	51.43	53.85	0.053
< 9.300	54.29	53.85	0.081
< 9.400	55.71	53.85	0.096
< 9.483	57.14	53.85	0.110
< 9.533	58.57	53.85	0.124
< 9.583	60.00	53.85	0.139
< 9.667	60.00	46.15	0.062
< 9.750	61.43	46.15	0.076
< 9.933	62.86	46.15	0.090
< 10.12	64.29	46.15	0.104
< 10.23	65.71	46.15	0.119
< 10.45	67.14	46.15	0.133
< 10.63	68.57	46.15	0.147
< 11.02	70.00	46.15	0.162
< 11.38	71.43	46.15	0.176
< 11.53	72.86	46.15	0.190
< 11.68	72.86	38.46	0.113
< 12.10	74.29	38.46	0.128
< 12.83	74.29	30.77	0.051
< 13.25	75.71	30.77	0.065
< 13.50	77.14	30.77	0.079
< 13.95	78.57	30.77	0.093

< 14.65	81.43	30.77	0.122
< 15.22	82.86	30.77	0.136
< 15.48	82.86	23.08	0.059
< 16.70	84.29	23.08	0.074
< 17.78	84.29	15.38	-0.003
< 18.07	85.71	15.38	0.011
< 18.88	87.14	15.38	0.025
< 20.25	87.14	7.692	-0.052
< 21.25	87.14	0	-0.129
< 21.87	88.57	0	-0.114
< 22.37	90.00	0	-0.100
< 23.07	91.43	0	-0.086
< 23.73	92.86	0	-0.071
< 25.23	94.29	0	-0.057
< 28.23	95.71	0	-0.043
< 29.88	97.14	0	-0.029

MBI: maximum blink interval
